# Pyrotinib‐associated acute kidney injury: A case report

**DOI:** 10.1002/cai2.38

**Published:** 2022-12-04

**Authors:** Kang Miao, Li Zhang, Xiaoyan Si

**Affiliations:** ^1^ Department of Pulmonary and Critical Care Medicine Peking Union Medical College Hospital, Chinese Academy of Medical Science & Peking Union Medical College Beijing China

**Keywords:** pyrotinib, HER2 TKIs, acute kidney injury and case report

## Abstract

Pyrotinib is a novel human epidermal growth factor receptor 2 (HER2) tyrosine kinase inhibitor (TKI). Previous studies of pyrotinib showed that it is mainly excreted through the gastrointestinal tract rather than the kidneys, with little effect on renal function. Here, we report a patient with HER2‐mutated nonsmall cell lung cancer who developed acute kidney injury (AKI) after receiving pyrotinib treatment. This case alerts clinicians to the adverse renal effects of HER2 TKIs, especially in patients with chronic kidney disease. However, tumor treatment should remain a priority in clinical practice. In this case, AKI induced by pyrotinib was reversible. Therefore, there is no need to restrict the use of HER2 TKIs due to concerns about possible nephrotoxicity.

## Graphical abstract

We report a case of a nonsmall cell lung cancer patient with human epidermal growth factor receptor 2 mutation developed acute kidney injury after pyrotinib treatment. He experienced three times renal function injuries that were clearly associated with pyrotinib challenges, which reminded clinicians to be aware of the nephrotoxicity of pyrotinib.

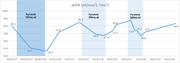

**Abbreviations**
AKI: acute kidney injury
ALK: anaplastic lymphoma kinase
CKD: chronic kidney disease
Cr: creatinine
CYP3A4: cytochrome P450 3A4 enzymes
EGFR: epidermal growth factor receptor
HER2: human epidermal growth factor receptor 2
ICIs: immune checkpoint inhibitors
NGS: next‐generation sequencing
NSCLC: non‐small cell lung cancer
OS: overall survival
PFS: progression‐free survival
TKI: tyrosine kinase

## 1 BACKGROUND

Currently, targeted therapy for nonsmall cell lung cancer (NSCLC) mainly focuses on classical mutations like epidermal growth factor receptor (EGFR) and anaplastic lymphoma kinase (ALK) [1]. Human epidermal growth factor receptor 2 (HER2) is one of the most common breast cancer driver genes, but is only mutated in about 2% of NSCLC patients [2]. Pyrotinib is a novel HER2 tyrosine kinase inhibitor (TKI) [3], with a median progression‐free survival (PFS) of 6.9 months and a median overall survival (OS) of 14.4 months in patients with NSCLC in phase II clinical trials [4]. Previous studies of pyrotinib showed that it is mainly excreted through the gastrointestinal tract rather than the kidneys, with little effect on renal function [5]. Here, we report a patient with HER2‐mutated NSCLC who developed acute kidney injury (AKI) following pyrotinib treatment.

## 2 CASE PRESENTATION

A 57‐year‐old man was diagnosed with squamous cell carcinoma (CT4N3M0, stage IIIc) in December 2019. Next‐generation sequencing (NGS) revealed HER2 exon 15 mutations (3.7%), with no other coexisting driver gene mutations. The patient had diabetes, hypertension, and grade 2 chronic kidney disease (CKD). Before anticancer treatment, creatinine (Cr) was 105 µmol/L (eGFR: 75.7 ml/min/1.73 m^2^) and protein excretion rate was 160 mg/24 h. Blood counts, urinalysis, and ultrasonography of the urinary system were unremarkable. The patient received four cycles of nab‐paclitaxel, cisplatin, and pembrolizumab as first‐line therapy with no significant toxicity but disease progression.

**Figure 1:**
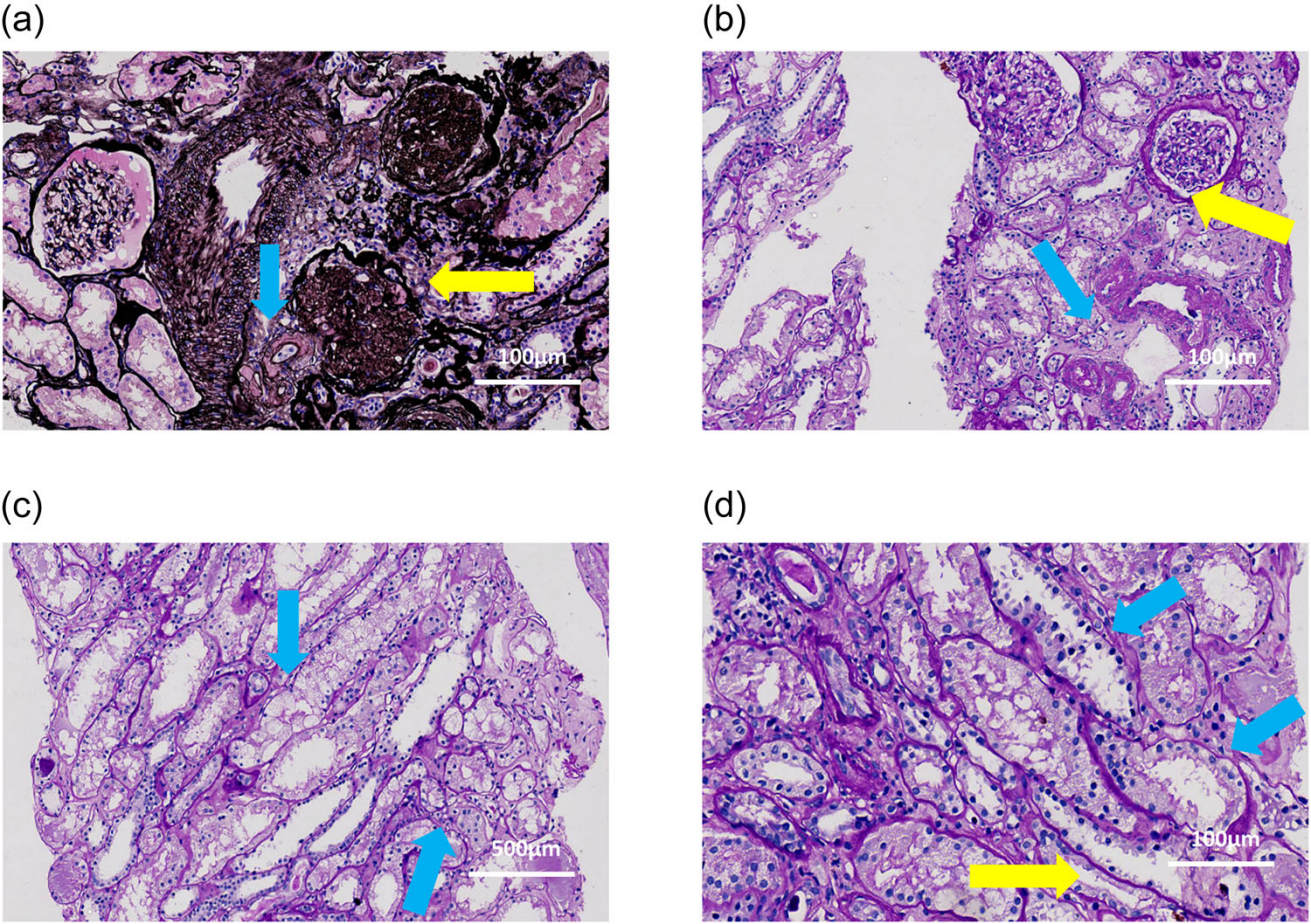
Pathological features detected by kidney biopsy. (a) Hyaline change of arterioles (blue arrow), glomerular sclerosis (yellow arrow) (periodic acid‐schiff‐methenamine stain, 200×); (b) thickening of arterioles (blue arrow), shrinking of glomerular capillary loop and fibrosis of Bowman's capsule (yellow arrow) (periodic acid‐schiff stain, 200×); (c) vacuolar degeneration of tubules (blue arrow) (periodic acid‐schiff stain, 400×); (d) regeneration of tubules (blue arrow), flattening of the tubular epithelium and detachment of brush border (yellow arrow) (periodic acid‐schiff stain, 200×).

**Figure 2:**
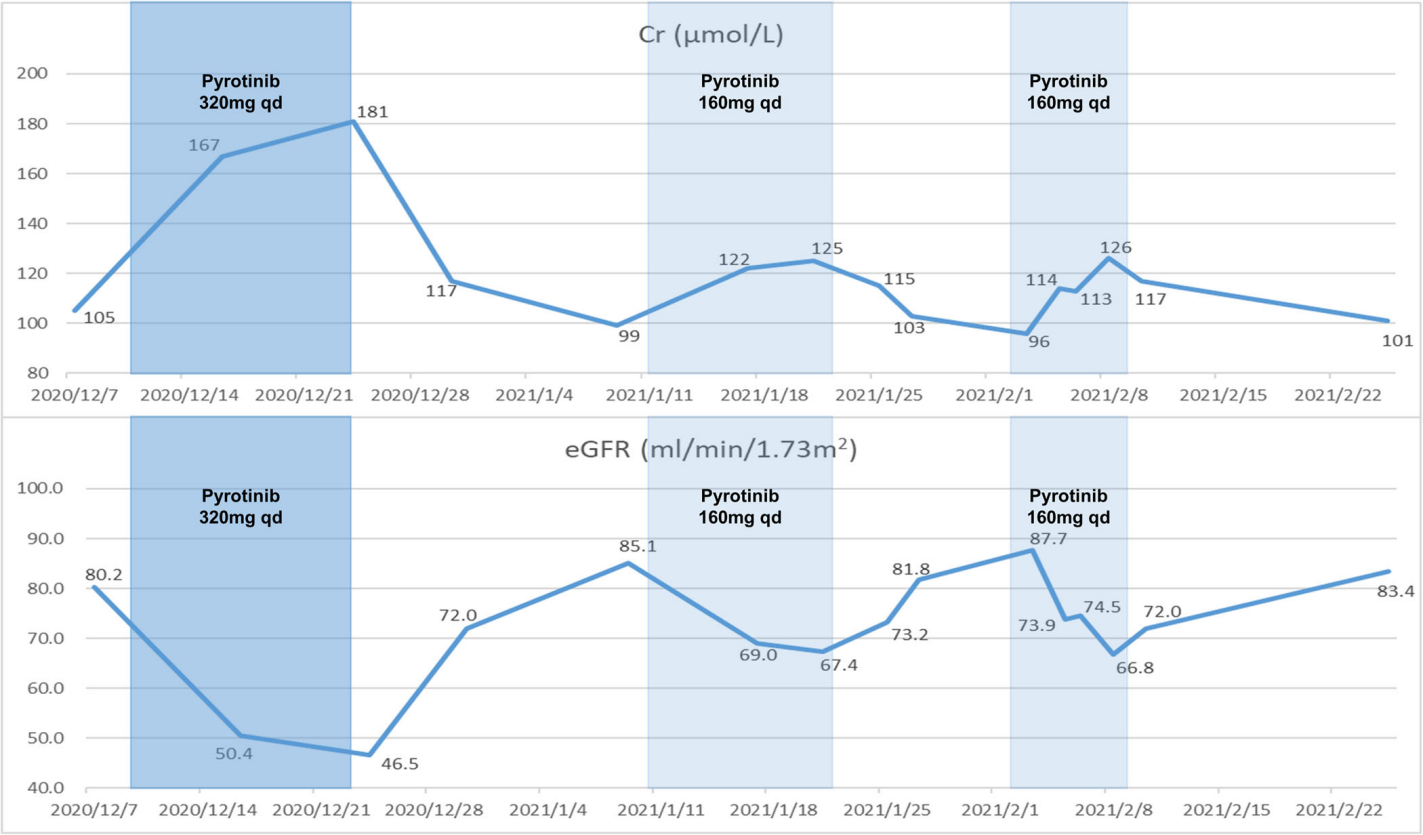
Temporal pattern of serum creatinine and eGFR related to pyrotinib administration

Due to the presence of HER2 mutations, he was started on pyrotinib 320 mg daily. After taking pyrotinib for 9 days, the Cr level increased to 181 µmol/L (eGFR: 46.5 ml/min/1.73 m^2^) and continued to deteriorate. Considering that the patient has CKD and the progressive decline of renal function, pyrotinib treatment was temporarily stopped. After stopping the drug, the Cr level gradually returned to the baseline level, and pyrotinib 160 mg was resumed. However, Cr levels rose again only 5 days later, thus a kidney biopsy was performed. The main pathological features under the microscope were glomerular ischemic sclerosis in nearly half of the glomeruli, thickening of vascular walls with hyaline deposits, focal tubular atrophy, and interstitial fibrosis with a small amount of inflammatory cell infiltration. These features were consistent with hypertensive renal damage. However, tubular vacuolar degeneration occurred in some renal tubules, which was represented by flattening tubular epithelium and detaching brush border, suggesting acute tubular injury (Figure 1). Six days after discontinuing of pyrotinib for the second time, the renal function returned to the baseline level again, and he received pyrotinib challenge for the third time. Unfortunately, after 5 days, the Cr level progressively increased to 126 µmol/L (eGFR: 66.8 ml/min/1.73 m^2^) and pyrotinib was terminated completely (Figure 2).

## 3 DISCUSSION

Studies have shown that pyrotinib is mainly metabolized by cytochrome P450 3A4 enzymes (CYP3A4) in liver and excreted through gastrointestinal tract [6]. After oral administration of _14_C‐labeled pyrotinib in healthy subjects, less than 2% of the radioactive material was excreted through urine, suggesting that pyrotinib had little effect on renal function [7]. Although no clear association between pyrotinib and nephrotoxicity has been reported in previous studies, we believe that the AKI in our case was most likely attributable to pyrotinib because of their close temporal correlation.

The etiology of acute renal injury can be divided into prerenal, renal, and postrenal factors. In terms of prerenal factors, the patient had hypertension and diabetes, but the condition was stable, and there was no strong correlation with AKI. In addition, radiological examination revealed no signs of urinary tract obstruction. Further consideration of renal factors, given that the patient had a clear history of antineoplastic drug administration, drug‐induced renal impairment should be considered first. Before pyrotinib, the patient received nab‐paclitaxel, cisplatin, and pembrolizumab. Cisplatin is a common nephrotoxic drug that causes renal tubular damage, oxidative stress, and vasoconstriction. Acute tubular necrosis was one of the main pathological manifestations of cisplatin‐induced AKI [8]. However, AKI occurred several months after complete cessation of cisplatin, suggesting that cisplatin was not the cause of AKI. Immune checkpoint inhibitors (ICIs), such as pembrolizumab, can also cause AKI, primarily manifested as tubulointerstitial nephritis, characterized by massive lymphocytic infiltration [9]. Unlike cisplatin, ICIs‐mediated AKI can occur 3–16 months after exposure because of activated T‐cell immunity. However, the renal pathology of this patient showed only a small amount of mononuclear cell infiltration, which was not consistent with the pathology of ICIs‐mediated AKI. AKI occurred during pyrotinib administration without concurrent use of other drugs. Diarrhea is a common side effect for pyrotinib and may affect renal function. However, in this case, the patient did not experience diarrhea or vomiting while taking pyrotinib. A study of a traditional HER2 monoclonal antibody (trastuzumab) concluded that it did not increase the risk of AKI when used in combination with chemotherapy (Incidence of kidney injury: trastuzumab +  chemotherapy vs. chemotherapy, 16% vs. 13%) [10]. Although the nephrotoxicity of pyrotinib has not been reported previously, our patient experienced three elevations of Cr levels that were apparently associated with three pyrotinib challenges. Additionally, all other medications that could cause kidney damage had been discontinued for several months. Therefore, in this case, pyrotinib most likely caused AKI by affecting the renal tubules.

## 4 CONCLUSION

This case alerts clinicians to the adverse effects of HER2 TKIs on the kidneys, especially in patients with CKD. However, oncology treatment should remain a priority in clinical practice. Our case also showed that AKI induced by pyrotinib is reversible, which means that there is no need to limit the use of HER2 TKIs due to concerns about possible renal adverse effects. In addition, more research on the renal effects of pyrotinib is needed in the future.

## AUTHOR CONTRIBUTIONS

**Kang Miao**: Data curation (lead); formal analysis (lead); software (lead); writing – original draft (lead); writing – review and editing (lead). **Li Zhang**: Conceptualization (equal); funding acquisition (equal); investigation (equal); methodology (equal); project administration (equal); resources (equal); supervision (equal); validation (equal); visualization (equal). **Xiaoyan Si**: conceptualization (equal); funding acquisition (equal); investigation (equal); methodology (equal); project administration (equal); resources (equal); supervision (equal); validation (equal); visualization (equal).

## CONFLICT OF INTEREST

The authors declare no conflict of interest.

## ETHICS STATEMENT

This report was performed in line with the principles of the Declaration of Helsinki. Human ethics application was approved by the Ethics Committee of Peking Union Medical Hospital (HS‐3350D).

## INFORMED CONSENT

The authors confirm receipt of written informed consent from patient to publish the images in Figure 1. The patient has given consent to submit the case report to the journal. The patient signed an informed consent for the publication of his data and photographs.

## Data Availability

The data that support the findings of this study are available from the corresponding author upon reasonable request.
